# Nano Silicon Modulates Chemical Composition and Antioxidant Capacities of Ajowan (*Trachyspermum ammi*) Under Water Deficit Condition

**DOI:** 10.3390/foods14010124

**Published:** 2025-01-03

**Authors:** Zahra Sobatinasab, Mehdi Rahimmalek, Nematollah Etemadi, Antoni Szumny

**Affiliations:** 1 Department of Horticulture, College of Agriculture, Isfahan University of Technology, Isfahan 84156-83111, Iran; z.sobatinasab@ag.iut.ac.ir (Z.S.); etemadin@iut.ac.ir (N.E.); 2 Department of Food Chemistry and Biocatalysis, Wrocław University of Environmental and Life Sciences, 50-375 Wrocław, Poland; antoni.szumny@upwr.edu.pl

**Keywords:** ajowan, nano silicon, drought stress, essential oil, phenol, flavonoid

## Abstract

Ajowan (*Trachyspermum ammi*) is an important spice in the food industry, as a well as a medicinal plant with remarkable antioxidant properties. In this study, its essential oil content, chemical composition, flavonoid content, phenolic content, and antioxidant capacity were evaluated under three irrigation regimes (50, 70, and 90% field capacity) and different amounts of nano silicon (0, 1.5, and 3 mM) in ten populations of ajowan. Based on the GC–MS analysis, thymol, carvacrol, *p*-cymene, and γ-terpinene were determined as the main components of the oil. The thymol content ranged from 34.16% in the Ardabil population (irrigation at 50% and nano silicon at 1.5 mM) to 65.71% in the Khorbir population (without nano silicon and irrigation at 50%). The highest phenolic content was in Khormo with irrigation at 90% and without nano silicon (172.3 mg TAE/g DW), while the lowest was found in Hamedan (irrigation at 50% and without nano silicon (7.2 mg TAE/g DW)). Irrigation at 50% and no nano silicon treatment led to an increase in total flavonoids in Ardabil (46.786 mg QUE/g DW). The antioxidant activity of ajowan was evaluated using the DPPH assay. Accordingly, the highest antioxidant capacity was observed in Khormo (irrigation at 90% without nano silicon; 4126 µg/mL). Moreover, the highest thymol content was observed in the Khorbir population with irrigation at 50% and without nano silicon treatment. Furthermore, correlation and principal component analysis (PCA) provide new insights into the production of ajowan from their substrates under nano silicon treatment and water deficit conditions. Finally, the results revealed information on how to improve the desired essential oil profile and antioxidant capacity of extracts for industrial producers.

## 1. Introduction

Ajowan, also known as *Trachyspermum ammi* (L.) Sprague, is a grassy and aromatic annual herbaceous herb with an erect and striate stem that has glabrous pubescent properties. It is a member of the medicinally significant Apiaceae family. This plant is an important spice because of its aroma and high thymol content in the seeds. The main components of ajowan oil are thymol, carvacrol, and *p*-cymene [1]. Different activities have been reported for ajowan, including antioxidant [2], antifungal [3], antibacterial [4], larvidical [5], insecticidal, and immune response [6].

Nowadays, agriculture is truly transforming thanks to new technologies such as nanotechnology. In the past ten years, the usage of nano fertilizers has enhanced productivity, decreased production costs, and furthermore decreased biotic and abiotic stresses, leading to the stability of production [7,8]. The key characteristic of these fertilizers is their greater solubility in comparison to other comparable non-nano fertilizers [9].

Since silicon (Si) provides structural cellular integrity, including for cell organelles, it may be able to assist plants in reacting to a water stress. In response to biotic or abiotic stressors, silicon nanoparticles (nSis) have shown promising effects in supporting healthy plant development, particularly crop yield [10,11,12].

Genetic and environmental factors can affect the chemical composition as well as biological activities of plant extracts [13,14]. Today, the appropriate management of water resources is a major concern, particularly for nations that are experiencing a water crisis. There are some reports regarding the effect of nano silicon for alleviating the drought stress problems in different plants including coriander [15], *Tanacetum parthenium* [16], and wheat [17]. However, there are no reports regarding the effect of nano silicon and water stress that assess the changes in essential oil components and biological activities of ajowan.

Thus, the objectives of this study were to, (1) for the first time, evaluate the variation in essential oil content and components in ten different ajowan species under three nano silicon amounts and three water stress levels; (2) to assess the total phenolic and flavonoid content and their antioxidant capacity; and (3) to use multivariate analyses for better interpretating metabolite changes and introducing elite genotypes.

## 2. Results and Discussion

### 2.1. Essential Oil Content

**Figure 1:**
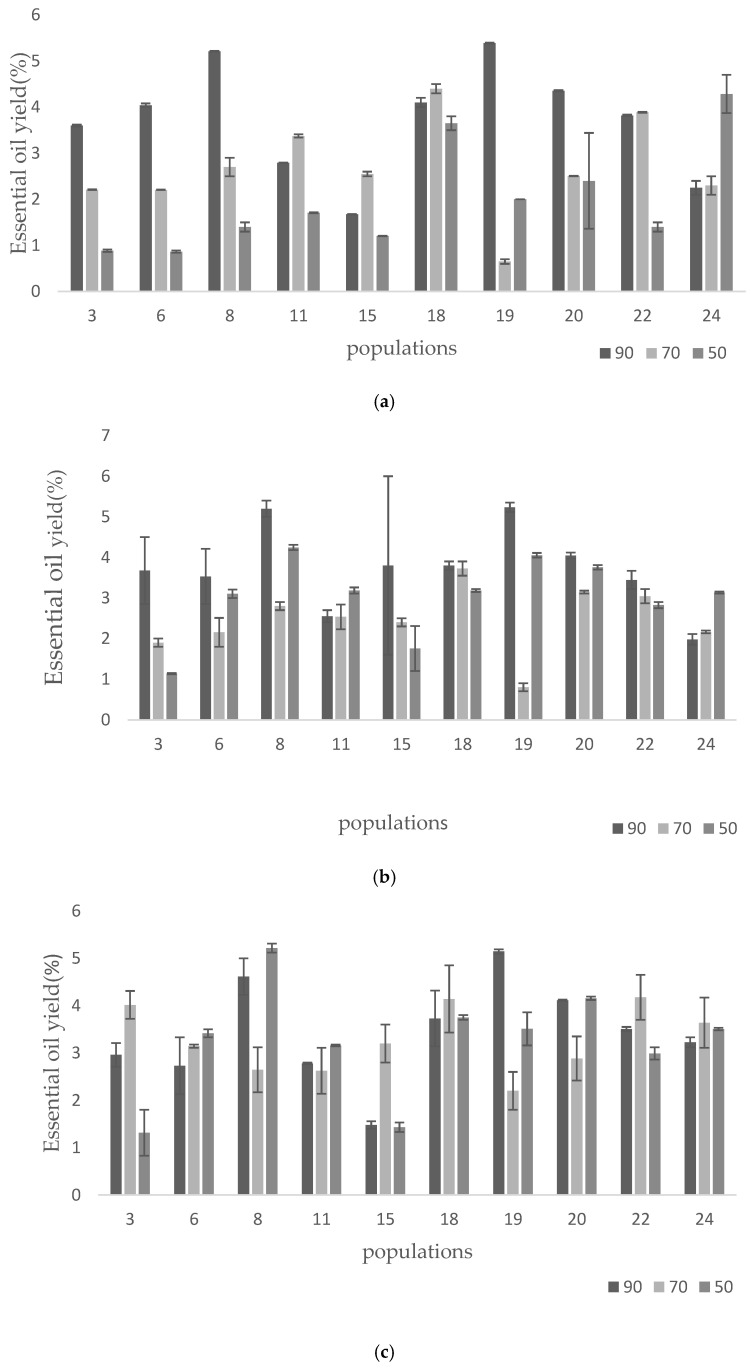
(**a**). Variation in essential oil yield between studied populations (x-axis) under control nano silicon (0 mM) and different irrigation regimes. (**b**). Variation in essential oil yield among studied populations (x-axis) under control nano silicon (1.5 mM) and different irrigation regimes. (**c**). Variation in essential oil yield between studied populations (x-axis) under control nano silicon (3 mM) and different irrigation regimes.

High variation was obtained in the studied populations and the studied treatments (Figure 1). The highest and lowest essential oil content was obtained in the populations of Esfahfo without nano silicon and irrigation at 90% (5.39%) and Arakkho with nano silicon at 3 mM and irrigation at 50% (5.21%), while the lowest amount belonged to Esfahfo with 70% irrigation (0.65%). In severe-stress conditions, the use of nano silicon in both concentrations led to an increase in the percentage of essential oil in all ten populations. Similar to the present research, some reports have also published on *Tanacetum partenium* [16] and *Cymbopogon flexuosus* [18]. The application of nano silicon complexes limited tissue dehydration and the development of oxidative damage under water deficit conditions and restored the growth and yield of plant essential oils [19].

In the present study, a 1.5 mM concentration of nano silicon along with 50% water stress condition led to the production of the highest oil content. Previous reports also highlighted the mechanism involved in improving the oil content by the application of nano silicon. SiO_2_NPs have a greater capacity to enter plant cells through their wall pores, which may help improve the physiology, growth, and generation of essential oils in plants [16]. Furthermore, the application of nano silicon decreases the tissue dehydration and oxidative damage under water deficit condition and, consequently, can restore the growth and essential oil yield [20]. Nano silicon can improve the essential oil production by its positive effect on water and nutrient uptake and source-sink potential [21].

Si accumulation can increase ROS generation and induce oxidative stress in plant cells, which are highly reactive and can induce lipid peroxidation, thereby causing damage to enzymes, proteins, and nucleic acids [22].

Si, along with lignin, can deposit in the dermal regions of cell walls, thickening the Casparian strips and blocking TE transport in plants. Si-induced changes in cell wall-binding properties could be essential in mitigating TE’s toxicity [23].

### 2.2. Essential Oil Composition

According to the GC–MS analysis, fourteen compounds were determined in the studied ajowan populations (Table 1, Table 2 and Table 3). The GC-MS chromatograms are illustrated in Figure S1. Consequently, thymol (34.16–65.71%), carvacrol (0.46–1.42%), *p*-cymene (11.87–26.41%), and γ-terpinene (14.11–32.14%) were the most abundant compounds. The lowest thymol content belonged to irrigation at 50% and nano silicon at 1.5 mM in Ardabil (34.16%), while the highest amount was obtained in 50% irrigation condition and without nano silicon in the Khorbir population (65.71%). The highest *p*-cymene content was associated with irrigation at 50% and 1.5 mM nano silicon in the Hamedan population (26.41%).

The lowest γ-terpinene content belonged to irrigation at 50% and nano silicon at 3 mM in the Khorbir population, while the highest amount was obtained in the Yazshah population irrigation at 50% and 1.5 mM nano silicon (Table 1).

There is limited research regarding the use of nano silicon as a component of essential oil from medicinal plants. In a similar investigation, a decrease in the number of essential oil components and significant changes in the amount and composition of the oil itself were observed in *Artmisia annua* [24].

In the present research, the use of nano silicon led to an increase in monoterpene accumulation that was consistent with that reported by [24]. The increase in the amount of monoterpene and decrease in sesquiterpenes can be attributed to different factors such as phenological stage, temperature, and type of stimulator or stress condition [25,26].

### 2.3. Total Phenolic Content (TPC) and Total Flavonoid Content (TFC)

The lowest TPC belonged to Hamedan, with irrigation at 50% and without nano silicon (7.2 mg TAE/g DW), while the highest TPC was observed in Khormo with irrigation at 90% and without nano silicon (172.3 mg TAE/g DW). The lowest and the highest TFC was obtained in Esfahfo (drought stress at 90% and without nano silicon), i.e., 0.755 mg QUE/g DW, and Ardabil (drought stress at 50% and without nano silicon), i.e., 46.786 mg QUE/g DW.

### 2.4. Antioxidant Capacity

The lowest and highest antioxidant activities were observed in Khormo (irrigation at 90% without nano si; 4126 µg/mL) and Hamedan (drought stress at 50% without nano si; 288.5 µg/mL), respectively. In the present study, the total phenolic (TPC) and flavonoid content (TFC) were dependent on the degree of water stress. Water stress can lead to an increase in reactive oxygen species, and therefore, higher amounts of antioxidants are required to compensate for stress conditions and increased tolerance [27]. Antioxidant activity is crucial in maintaining the balance between the production and scavenging of free radicals [28]. Furthermore, an increase in TPC under drought stress is highly correlated with the production and distribution of different antioxidants in the plant and the duration and intensity of stress [29,30].

Fischer et al. [29] assessed the correlation of TPC and antioxidant activity based on antioxidant activity under drought and normal conditions. They revealed that, under drought stress conditions, there was a weak correlation between the results determined by the antioxidant activity and the TPC methods, while under normal field conditions, a better correlation was observed that was in agreement with that obtained in the present research. This might be due to different phenolic compounds and their functional variations under different environmental conditions [30].

Most of the polyphenols can be upregulated with increasing drought stress [31]. In contrast, the higher levels of flavonols were indicated under extreme drought stress in Arabidopsis. The response of flavonoids to drought stress has been investigated as variable, and the severity and duration of drought stress may have a significant impact on the types, quantities, and localization of flavonoids in response to various levels of water shortage [32].

The improvement in the antioxidant capacity due to nano si is one of the mechanisms for plant protection against oxidant stresses. Moreover, the accumulation of flavonoids and phenolic acids is essential to reduce the negative effects of drought stress in plants; higher concentrations of nano compounds, such as nano si, can alleviate the negative effects of water stress. Moreover, flavonoids have been considered as health-beneficial compounds, and nano si can protect these valuable components from being lost during stresses. Flavonoid production in the cytoplasm can detoxify the harmful H_2_O_2_ molecules produced during drought stress [33]; the flavonoid levels also increased and demonstrated that a water deficit condition had an effect on flavonoid accumulation, possibly by regulating hormone metabolism [34].

### 2.5. Correlation Analysis

For better interpretation of the results, correlation analysis between compounds was performed. Accordingly, thymol showed a high negative correlation with *p*-cymene (−0.81517) and γ–terpenene (−0.713). Thymol is produced by the aromatization of γ-terpinene to *p*-cymene followed by the hydroxylation of p-cymene. Thus, in the present research, thymol production from its substrates was induced in response to nano silicon and drought stress. Accordingly, decreases in two substrates, viz. *p*-cymene and γ–terpenine, led to an elevation in thymol production. Furthermore, based on the results, in most cases, the increase in nano silicon leads to a decrease in the main components of the ajowan oil (Table 4, Table 5 and Table 6).

**Figure 2:**
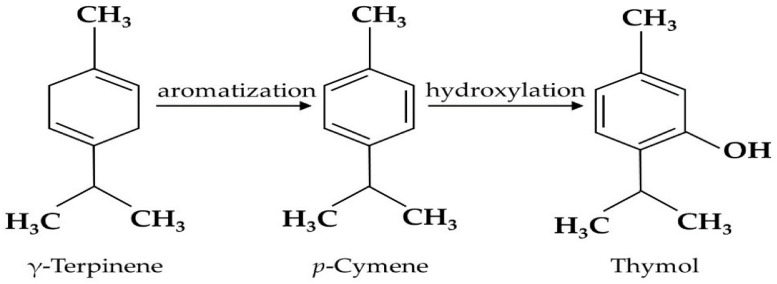
The biosynthesis of thymol, γ-terpinene, and *p*-cymene [35].

Thus, it can be suggested that nano silicon can lead to a decrease in thymol content by providing a surface in the epidermis to protect tissues from water loss during a water deficit condition. In contrast, in the absence of nano silicon, the release of compounds can be much easier, as the essential oil in Apiaceae is mainly located in secretary sacs and channels in the parenchyma (1), and consequently, the degradation of its structure through water stress and the absence of nano silicon can be an efficient way to elevate the essential oil content (Figure 2).

### 2.6. Principal Component Analysis (PCA)

**Figure 3:**
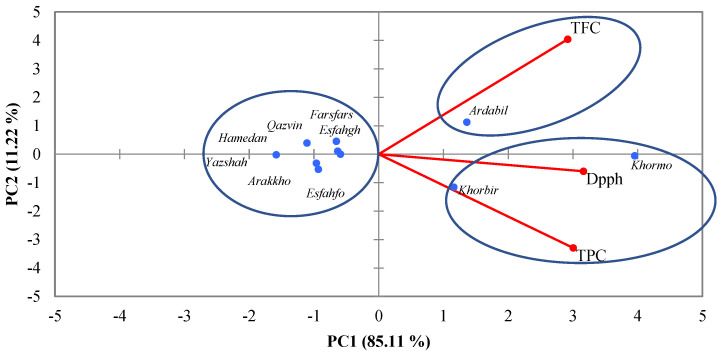
The PCA plots illustrate the interaction effects of drought stress at 90% on TPC, TFC, and DPPH in populations of *Trachyspermum ammi*.

On the basis of PCA, three biplots were designed under three water deficit conditions. In the normal irrigation regime (90% FC), the cultivars were divided into three groups. In the first group, the Khormo and Khorbir populations possessed the highest antioxidant capacity and TPC, and in the second group, Ardabil had a higher correlation in terms of TFC, and other cultivars showed no correlation with any of the measured traits, and they were classified in a separate group (Figure 3).

**Figure 4:**
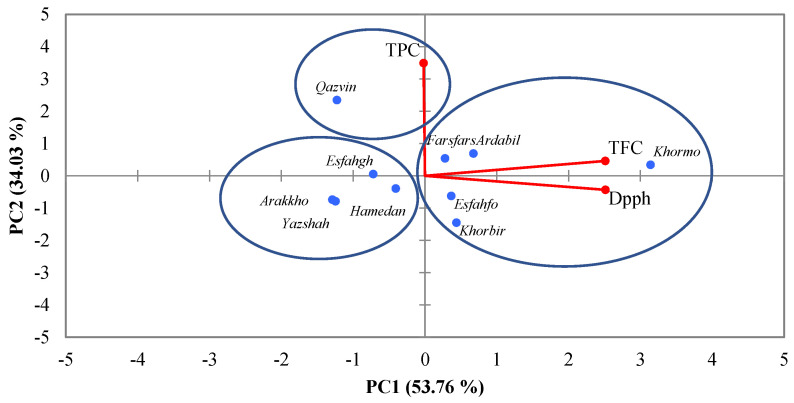
The PCA plots illustrate the interaction effects of drought stress at 70% on TPC, TFC, and DPPH in populations of *Trachyspermum ammi*.

In the medium-stress condition (70% irrigation) with the application of silicon at a concentration of 1.5 mM, there was a positive correlation between the traits of antioxidant capacity and total flavonoid content, and there was no relation with TPC. The Qazvin population showed the highest amount of TPC, and the third group was not included in any of the traits (Figure 4).

**Figure 5:**
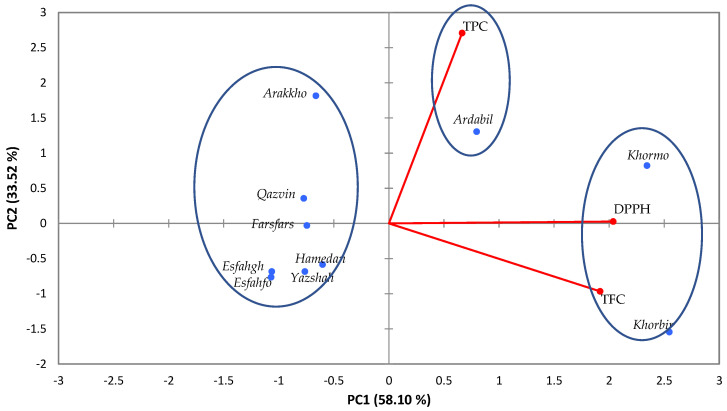
The PCA plots illustrate the interaction effects of drought stress at 50% on TPC, TFC, and DPPH in populations of *Trachyspermum ammi*.

Under severe-stress conditions, specifically with 50% irrigation and a concentration of 3 mM nano silicon, we observed a positive correlation between DPPH and TFC, while no correlation was found with TPC. The cultivars were divided into three groups. The first group was the Khormo and Khorbir populations in terms of antioxidant properties. The total flavonoid content (TFC) revealed higher levels in the second sample of the Ardabil population with the TPC trait. Other cultivars had no significant correlation with any of the measured traits and were grouped in a separate group (Figure 5).

## 3. Materials and Methods

### 3.1. Plant Materials

Ten Iranian ajowan seeds were obtained from the Research Institute of Forests, Range, and Watershed Management Organization’s gene bank. Prof. Valiolah Mozaffarian used Flora Iranica [36] for plant identification. The botanical characteristics of the studied plants are shown in Table 7.

### 3.2. Experiment Design

The seeds were planted on May 10, 2023, on the research farm of the Isfahan University of Technology, Isfahan province, Iran (32°59′ N and 50°24′ E), at an altitude of 1900 m above sea level. A factorial randomized complete block design, with three replications, was applied for the experiment. The three levels of irrigation regimes were deficit irrigation (which met 50% and 70% of the irrigation requirement) and full irrigation (90% of the irrigation requirement). Clay soil was applied with pH = 7.38 and EC = 3.25 ds/m. The seeds were grown in the pot with a width and height of 25 cm. In order to apply irrigation treatments, the method described in [37] was applied.

### 3.3. Nano Silicon Production

The nanochelated fertilizers, which were ground to a 2 g L^−1^ concentration, were used for the foliar spraying of the subplots. The nanochelated fertilizers contained 2% of chelated silicon. The initial round of foliar fertilizer was applied during the tillering growth stage, with subsequent treatments spaced out at 15 days. The non-chelated silicon was purchased from Khazra Company, Tehran, Iran, with a patent of USPTO [17].

The chelated nano fertilizers were created by dissolving silicon components in water and shaking the mixture. After the compound had fully dissolved in the water, organic acid was added and allowed to dissolve entirely in the mixture. The initiator was added at this point to enable the creation of nuclei. When the nuclei generation reached the appropriate amount after 8–10 h, the capping agent was used to control the nuclei generation. The solution was then allowed to stabilize in the lab setting for a duration of six hours. After their deposition, the nanoparticles were separated by filtering and dried at 70 °C in an oven.

### 3.4. Essential Oil Distillation

The harvested mature seeds were firstly powdered. Then, 50 g of this seed powder was used for hydro-distillation for 6 h using a Clevenger-type apparatus. The EO yield was calculated based on the following formula [38]:

EO yield (%) = volume of EO obtained (mL) × 100/mass of dry matter (g)

### 3.5. GC–MS Analysis

An Agilent 7890 gas chromatograph (Agilent Technologies, Palo Alto, CA, USA) was applied to analyze the volatile components of the ajowan oil. HP-5MS with a 5% phenylmethylsiloxane capillary column (30 m, 0.25 mm, and a film thickness of 0.25 m) was used in this study. Furthermore, helium was applied as the carrier gas for the present study, with a split ratio of 1:20 and a flow rate of 2 mL min^−1^. The oven was preheated to 60 °C for three minutes and then ramped up to 120 °C at 3 °C per minute. Finally, it was increased to 300 °C at 15 °C per minute. The injector temperature was maintained at 300 °C. An Agilent 5975 C mass detector (Agilent Technologies, Palo Alto, CA, USA) was applied. The scanning conditions comprised 39–400 m/z, 200 °C, and an electron ionization of 70 eV. The injection volume was set at 1 µL of 0.1% EO solution in cyclohexane.

### 3.6. Identification of Essential Oil Constituents

The following methods were used to determine the constituents of essential oils: (a) the mass spectra of unknown compounds with spectra presented in NIST 17 (National Institute of Standards and Technology), Wiley 275. L, and the literature data; (b) logarithmic retention indices (RI) in relation to a series of n-alkanes (C8–C24) with data published in the NIST17 database and Adams and Sparkman [39], *identification of essential oil components by gas chromatography/mass spectrometry* (Vol. 456, pp. 544–545), Carol Stream: Allured Publishing Corporation; and (c) standards’ retention periods. For MS Search, the minimum match value was 90%. On the basis of the peaks of GC–MS chromatograms, the percentage of identified chemicals in EOs was calculated.

### 3.7. Methanolic Extract and Total Phenolic Content

The total phenolic content (TPC) was determined using the technique explained by Gharibi et al. [40]. For this purpose, eight grams of the dried material was extracted using 200 mL of 80% methanol and a shaker operated at 150 rpm for twenty-four hours at 25 °C. The procedures were then repeated three times after the extracts had been screened. The reaction mixture was made up of 2.5 mL of Folin–Ciocalteu reagent, 0.5 mL of extract, and 2 mL of sodium carbonate (7.5%). Ultimately, the absorbance at 765 nm was determined, and the tannic acid equivalent per gram dry weight of TPC was reported.

### 3.8. Total Flavonoid Content (TFC)

The aluminum chloride colorimetric method was applied for the determination of TFC [41]. First, 75 µL of the NaNO_2_ solution (5%) was mixed with 125 µL of the extract. The blending was performed for six minutes. Then, 150 µL of AlCl_3_ (10%) was added and incubated for 5 min, and finally, 750 µL of NaOH (1 M) was added. The absorbance of the pink extract was evaluated at 510 nm. TFC was presented in mg of quercetin equivalents (QEs) per gram of the extract.

### 3.9. Antioxidant Activity

The DPPH assay was used as the method for evaluating the antioxidant capacity in the studied ajowan populations. The procedure was performed based on the method described by Tohidi et al. [42]. Consequently, 0.1 mL of plant extracts was combined with 5 mL of 0.1 mM methanolic DPPH solution at various concentrations of 50, 100, 300, and 500 ppm. Absorbances were then evaluated at 517 nm. In addition, BHT was employed as a synthetic antioxidant. Lastly, the EC_50_ value for antioxidant capacity was applied.

### 3.10. Statistical Analysis

The mean data of three replications per treatment for each trait were analyzed by combined ANOVA using SAS 9.4. The mean values of experimental treatments were compared by the LSD test at the 5% level. Every test was run in three replicates. The collected data were reported as means with standard deviation (SD). The Statgraphics Software (ver. 18) and SAS JMP version 11 were used for cluster analysis and principal component analysis (PCA).

## 4. Conclusions

This study provides new insights into the effects of nano silicon and water deficit stress on the secondary metabolite variation in ten populations of ajowan. Regarding the essential oil content, the Esfahfo population produced the highest yield without nano silicon and with irrigation at 90%. Moreover, the highest thymol content was observed in the Khorbir population with irrigation at 50% and without nano silicon treatment. Furthermore, in the present study, the highest phenolic and flavonoid content was obtained in 90% and 50% water stress condition without silicon. Finally, the results of this research can introduce the best conditions and elite ajowan genotypes for providing the best metabolites in appropriate water conditions.

## Figures and Tables

**Table 1:** Ajowan essential oil composition with irrigation at 50%.

		Carvacrol	Thymol	Pulegone	Terpinene 4-ol	β- Thujone	*Cis*- Sabinenehydrate	γ- Terpinene	*p*- Cymene	α-Terpinene	Myrcene	β-Pinene	Sabinene	α-Pinene	α-Thujene	Total
	RI^exp^/RI^lit^	1301/129	1290/129	1246/1237	1181/117	1110/1114	1068/1070	1057/1060	1025/1024	1017/1017	996/99	979/979	972/97	941/937	927/92	
Si	gen															
Si 0	p3	1.42	65.71	0.29	0.23	0.35	0.27	16.18	12.89	0.15	0.25	0.44	0.16	0.08	0.24	98.66
	P6	0.63	40.52	0.16	0.26	3.98	0.21	16.98	16.56	0.54	0.86	0.85	0.26	0.67	1.14	83.62
	p8	0.94	42.07	0.08	0.18	1.35	0.01	26.13	25.09	0.62	0.65	1.61	0.37	0.27	0.63	100
	p11	1.01	38.49	0.15	0.21	0.7	0.17	31.11	25.13	0.62	0.64	0.34	0.42	0.23	0.78	100
	p15	0.61	40.34	0.09	0.2	0.61	0	31.11	24.35	0.53	0.61	0.41	0.35	0.15	0.54	99.9
	p19	0.46	46.38	0	0.16	0.62	0.15	26.71	23.25	0.44	0.35	0.52	0.27	0.16	0.53	100
	p20	0.71	43.89	0.18	0.42	0.65	0.21	26.09	24.52	0.54	0.69	0.68	0.36	0.18	0.66	99.78
	p22	0.6	41.39	0	0.29	15.12	0.17	20.58	19.61	0.46	0.49	0.34	0.31	0.11	0.53	100
	p18	0.62	50.98	0.48	0	0.84	0	25.06	19.08	0.23	0.49	1.37	0.23	0.19	0.43	100
	p24	0.61	44.31	0.17	0.28	0.69	0.28	26.81	21.66	0.65	0.76	0.29	0.42	0.17	0.64	100
	p3	1.41	64.13	0.29	0.25	0.37	0.25	17.11	12.38	0.15	0.24	0.48	0.17	0.09	0.25	97.57
	p6	0.54	65.57	0.24	0.28	2.39	0.23	15.09	12.11	0.51	0.61	0.69	0.22	0.4	1.12	100
	p8	0.58	47.99	0.016	0.22	6.24	0.014	19.04	21.68	0.59	0.8	1.73	0.29	0.25	0.56	100
	p11	1.16	37.59	0.18	0.24	0.85	0.11	30.19	26.41	0.64	0.83	0.34	0.43	0.21	0.82	100
Si 1.5	p15	0.56	40.21	0.09	0.23	0.65	0	32.14	23.66	0.51	0.59	0.39	0.35	0.11	0.51	100
	p19	0.45	47.12	0	0.17	0.65	0.1	25.34	23.87	0.45	0.44	0.53	0.23	0.17	0.48	100
	p20	0.82	41.12	0.07	0.27	0.54	0.13	27.11	23.76	0.48	0.71	0.64	0.34	0.19	0.67	96.85
	p22	0.61	34.16	0	0.29	20.94	0.15	20.08	20.91	0.51	0.69	0.46	0.36	0.3	0.54	100
	p18	0.62	50.13	0.49	0	0.85	0	25.18	18.02	0.25	0.48	1.13	0.24	0.17	0.42	97.98
	p 24	0.63	43.98	0.16	0.28	0.64	0.25	25.81	21.55	0.62	0.75	0.31	0.44	0.18	0.63	96.23
	p3	1.31	63.76	0.25	0.24	0.35	0.25	16.21	11.87	0.12	0.21	0.31	0.19	0.05	0.22	95.34
	p6	0.54	65.19	0.23	0.31	2.61	0.22	14.11	13.31	0.51	0.22	0.76	0.26	0.69	1.04	100
	p8	0.55	49.21	0.02	0.16	3.61	0.07	23.11	20.15	0.59	0.41	1.09	0.31	0.28	0.44	100
	p11	1.16	37.54	0.19	0.26	0.86	0.7	31.86	24.22	0.56	0.79	0.31	0.39	0.31	0.78	99.93
Si 3	p15	0.63	39.87	0.016	0.2	0.63	0	30.13	23.09	0.49	0.56	0.39	0.33	0.13	0.51	96.976
	p19	0.43	46.31	0	0.18	0.61	0.1	23.85	22.88	0.39	0.69	0.44	0.31	0.21	0.61	97.01
	p20	0.79	40.18	0.09	0.21	0.61	0.15	26.65	22.18	0.41	0.49	0.56	0.35	0.15	0.51	93.33
	p22	0.47	37.14	0	0.24	21.11	0.17	22.11	16.61	0.36	0.51	0.41	0.21	0.18	0.48	100
	p18	0.61	51.12	0.51	0	0.81	0	23.1	17.88	0.21	0.47	1.09	0.27	0.21	0.39	96.67
	p24	0.65	42.77	0.14	0.25	0.59	0.27	24.31	20.46	0.56	0.69	0.27	0.48	0.14	0.59	92.17

**Table 2:** Ajowan essential oil composition with irrigation at 70%.

		Carvacrol	Thymol	Pulegone	Terpinene 4-ol	β-Thujone	Cis-Sabinenehydrate	γ-Terpinene	*p*-Cymene	α-Terpinene	Myrcene	β-Pinene	Sabinene	α-Pinene	α-Thujene	Total
	RI^exp^/RI^lit^	1301/1299	1290/12	1246/12	1181/11	1110/111	1068/1070	1057/1060	1025/102	1017/1017	996/991	979/979	972/97	941/937	927/929	
Si	gen															
Si 0	P3	0.51	51.49	0	0.48	0.75	0	26.13	18.26	0.45	0.53	0.63	0.25	0.15	0.37	100
	P6	0.81	62.21	0	0.18	0.53	0	19.41	15.12	0.15	0.33	0.9	0.14	0.01	0.21	100
	P8	0.59	51.33	0.28	0.16	0.64	0	25.55	18.13	0.35	0.56	1.33	0.35	0.19	0.46	99.92
	P11	0.71	53.65	0.44	0.27	0.49	0.23	23.1	19.3	0.27	0.41	0.4	0.29	0.13	0.31	100
	P15	0.62	53.3	0.51	0.32	0.66	0	23.41	19.23	0.21	0.51	0.31	0.35	0.16	0.41	100
	P19	0.62	52.31	0.71	0.32	0.56	0	23.5	20.31	0.18	0.39	0.52	0.23	0.14	0.21	100
	P20	0.5	47.91	0.35	0.29	0.72	0	26.81	20.17	0.35	0.56	0.43	0.32	0.15	0.45	99.01
	P22	0.61	47.13	0.35	0.29	0.71	0	25.18	20.19	0.35	0.59	0.43	0.32	0.14	0.45	96.74
	P18	0.54	50.19	0.12	0.28	0.67	0	19.34	18.21	0.38	0.53	0.41	0.28	0.12	0.26	91.33
	P24	0.07	40.87	0	0	0.47	0	21.35	20.97	0.31	0.26	0.14	0.16	0	0.28	84.88
	p 3	0.55	53.16	0	0.44	0.76	0	25.24	17.61	0.39	0.48	0.61	0.27	0.16	0.33	100
	p 6	0.81	63.16	0.21	0.21	0.54	0	19.23	14.84	0.14	0.32	0.11	0.18	0.011	0.22	99.98
	p8	0.52	52.17	0.31	0.17	0.61	0	24.65	17.24	0.36	0.52	1.23	0.36	0.18	0.46	98.78
	p 11	0.83	54.14	0.52	0.29	0.69	0.21	21.63	19.66	0.28	0.46	0.51	0.33	0.14	0.31	100
Si 1.5	p 15	0.52	51.46	0.68	0.31	0.72	0	23.17	19.31	0.19	0.52	0.36	0.35	0.17	0.45	98.21
	p19	0.61	51.87	0.36	0.29	0.72	0	23.44	20.42	0.19	0.47	0.61	0.24	0.15	0.33	99.7
	p20	0.46	46.77	0.39	0.21	0.74	0	25.99	20.31	0.36	0.54	0.41	0.33	0.16	0.46	97.13
	p 22	0.65	42.81	0.14	0.27	0.73	0	24.86	20.22	0.37	0.56	0.41	0.33	0.15	0.45	91.95
	p 18	0.55	53.16	0	0.24	0.68	0	19.23	18.31	0.35	0.54	0.32	0.25	0.13	0.27	94.03
	p 24	0.09	41.22	0	0	0.48	0	22.19	20.99	0.32	0.27	0.15	0.17	0	0.28	86.16
	p3	0.57	52.19	0	0.36	0.68	0	30.22	13.69	0.48	0.43	0.55	0.29	0.17	0.37	100
	p6	0.82	62.18	0	0.22	0.56	0	20.11	13.81	0.15	0.31	0.12	0.19	0.019	0.23	98.71
	p8	0.48	49.17	0	0.19	0.63	0	23.88	16.22	0.37	0.51	1.25	0.35	0.19	0.47	93.71
	p 11	0.83	54.22	0.24	0.21	0.74	0	23.78	12.23	0.29	0.57	0.53	0.33	0.15	0.35	94.47
	p15	0.53	52.19	0.23	0.27	0.67	0	22.97	18.76	0.16	0.46	0.37	0.37	0.18	0.41	97.57
Si 3	p19	0.62	52.17	0.11	0.22	0.69	0	22.76	20.17	0.16	0.53	0.58	0.23	0.16	0.31	98.71
	p20	0.47	47.25	0.25	0.21	0.73	0	26.11	20.19	0.37	0.49	0.38	0.35	0.17	0.45	97.42
	p 22	0.66	40.13	0.09	0.23	0.74	0.008	23.87	20.18	0.38	0.51	0.37	0.34	0.16	0.43	88.09
	p18	0.56	50.22	0	0.21	0.69	0	18.87	17.87	0.33	0.53	0.37	0.26	0.14	0.27	90.32
	p24	0.011	40.67	0	0	0.44	0	23.09	19.87	0.33	0.29	0.14	0.18	0	0.29	85.31

**Table 3:** Ajowan essential oil composition with irrigation at 90%.

		Carvacrol	Thymol	Pulegone	Terpinene 4-ol	**β-** **Thujone**	Cis- Sabinenehydrate	γ-Terpinene	*p*-Cymene	α- Terpinene	Myrcene	β-Pinene	Sabinene	α-Pinene	α-Thujene	Total
	* RI^exp^/RI^lit^	1301/1299	1290/1291	1246/1237	1181/1177	1110/1114	1068/1070	1057/1060	1025/1024	1017/1017	996/991	979/979	972/974	941/937	927/929	
si	gen															
Si 0	p 3	0.51	52.49	0	0.48	0.75	0	26.13	17.26	0.45	0.54	0.63	0.25	0.15	0.36	100
	p 6	0.84	61.45	0	0.37	0.64	0	19.59	15.13	0.15	0.41	0.95	0.19	0.013	0.24	99.973
	p 8	0.59	51.33	0.28	0.16	0.64	0	25.55	18.13	0.35	0.56	1.33	0.35	0.19	0.46	99.92
	p 11	0.75	54.86	0.44	0.27	0.75	0.23	22.18	18.3	0.27	0.59	0.56	0.31	0.11	0.38	100
	p 15	0.62	53.3	0.57	0.32	0.76	0	23.49	19.23	0.21	0.41	0.38	0.31	0.16	0.24	100
	p 19	0.65	52.31	0.63	0.32	0.71	0	23.39	20.31	0.18	0.31	0.53	0.21	0.13	0.32	100
	p 20	0.5	47.91	0.35	0.29	0.72	0	26.81	20.17	0.35	0.56	0.43	0.32	0.15	0.45	99.01
	p 22	0.61	47.13	0.35	0.29	0.71	0	25.18	20.19	0.35	0.59	0.43	0.32	0.14	0.45	96.74
	p18	0.54	50.19	0.12	0.28	0.67	0	19.34	18.21	0.38	0.53	0.41	0.28	0.12	0.26	91.33
	p 24	0.07	40.87	0	0	0.47	0	21.35	20.97	0.31	0.26	0.14	0.16	0	0.28	84.88
	p 3	0.53	53.21	0	0.44	0.69	0	25.24	17.52	0.43	0.55	0.65	0.27	0.16	0.31	100
	p 6	0.81	63.16	0.21	0.21	0.54	0	19.23	14.84	0.14	0.32	0.11	0.18	0.011	0.22	99.981
	p 8	0.52	52.17	0.31	0.17	0.61	0	24.65	17.24	0.36	0.52	1.23	0.36	0.18	0.46	98.78
	p 11	0.76	54.21	0.53	0.29	0.62	0.21	21.66	19.74	0.28	0.51	0.41	0.33	0.14	0.31	100
Si 1.5	p 15	0.52	51.46	0.68	0.31	0.72	0	23.17	19.31	0.19	0.52	0.36	0.35	0.17	0.45	98.21
	p19	0.61	51.87	0.36	0.29	0.72	0	23.44	20.42	0.19	0.47	0.61	0.24	0.15	0.33	99.7
	p 20	0.46	46.77	0.39	0.21	0.74	0	25.99	20.31	0.36	0.54	0.41	0.33	0.16	0.46	97.13
	p22	0.65	42.81	0.14	0.27	0.73	0	24.86	20.22	0.37	0.56	0.41	0.33	0.15	0.45	91.95
	p 18	0.55	53.16	0	0.24	0.68	0	19.23	18.31	0.35	0.54	0.32	0.25	0.13	0.27	94.03
	p 24	0.09	41.22	0	0	0.48	0	22.19	20.99	0.32	0.27	0.15	0.17	0	0.28	86.16
	p 3	0.57	52.19	0	0.36	0.63	0	31.27	12.66	0.44	0.54	0.51	0.29	0.17	0.37	100
	p 6	0.82	62.18	0	0.22	0.56	0	20.11	13.81	0.15	0.31	0.12	0.19	0.019	0.23	98.719
	p 8	0.48	49.17	0	0.19	0.63	0	23.88	16.22	0.37	0.51	1.25	0.35	0.19	0.47	93.71
	p 11	0.83	54.22	0.24	0.21	0.74	0	23.78	12.23	0.29	0.57	0.53	0.33	0.15	0.35	94.47
Si 3	p 15	0.53	52.19	0.23	0.27	0.67	0	22.97	18.76	0.16	0.46	0.37	0.37	0.18	0.41	97.57
	p 19	0.62	52.17	0.11	0.22	0.69	0	22.76	20.17	0.16	0.53	0.58	0.23	0.16	0.31	98.71
	p 20	0.47	47.25	0.25	0.21	0.73	0	26.11	20.19	0.37	0.49	0.38	0.35	0.17	0.45	97.42
	p 22	0.66	40.13	0.09	0.23	0.74	0.008	23.87	20.18	0.38	0.51	0.37	0.34	0.16	0.43	88.098
	p 18	0.56	50.22	0	0.21	0.69	0	18.87	17.87	0.33	0.53	0.37	0.26	0.14	0.27	90.32
	p 24	0.011	40.67	0	0	0.44	0	23.09	19.87	0.33	0.29	0.14	0.18	0	0.29	85.311

* RI^exp^ (experimental retention index) and RI^lit^ (retention index of literature) based on the HP-5MS column.

**Table 4:** Correlation between compounds at 50% drought stress.

	Carvacrol	Thymol	Pulegone	Terpinene 4-ol	β- Thujone	Cis- Sabinenehydrate	γ- Terpinene	*p*- Cymene	γ- Terpinene	Myrcene	β-Pinene	Sabinene	α-Pinene	α- Thujene
Carvacrol	1													
Thymol	0.291832	1												
Pulegone	0.309017	0.497146	1											
Terpinene 4-ol	0.151771	−0.07908	−0.48411	1										
β-Thujone	−0.27384	−0.3128	−0.37957	0.211713	1									
*Cis*-Sabinenehydrate	0.421066	0.086727	0.018869	0.51061	−0.01274	1								
γ-Terpinene	−0.0851	−0.71397	−0.18401	−0.16174	−0.28176	−0.11484	1							
*p*-Cymene	−0.21786	−0.80517	−0.45068	0.01045	−0.1345	−0.18267	0.865208	1						
α-Terpinene	−0.3334	−0.57931	−0.54416	0.440313	0.043745	0.079382	0.418456	0.636256	1					
Myrcene	−0.21707	−0.69176	−0.25478	0.23473	0.062461	0.083156	0.436286	0.604864	0.709356	1				
β-Pinene	−0.19221	0.159299	0.257607	−0.47244	−0.04754	−0.48676	−0.20548	−0.03592	−0.0372	0.025792	1			
Sabinene	−0.11314	−0.68547	−0.29929	0.279133	−0.10485	0.125407	0.649693	0.731821	0.776564	0.691353	−0.24639	1		
α-Pinene	−0.26191	0.084096	0.027498	0.182037	0.1009	0.157086	−0.3696	−0.20292	0.35143	0.197537	0.277127	−0.0322	1	
α-Thujene	−0.26278	−0.14945	−0.11993	0.39239	−0.02016	0.246324	−0.0415	0.10252	0.63889	0.523675	0.000	0.312457	0.807838	1

**Table 5:** Correlation between compounds at 70% drought stress.

	Carvacrol	Thymol	Pulegone	Terpinene 4-ol	β- Thujone	Cis- Sabinenehydrate	γ- Terpinene	p- Cymene	α- Terpinene	Myrcene	β-Pinen e	Sabinene	α- Pinene	α- Thujene
Carvacrol	1													
Thymol	0.734661	1												
Pulegone	0.279629	0.12061	1											
Terpinene 4-ol	0.548494	0.37583	0.275303	1										
β-Thujone	0.369947	−0.02658	0.154703	0.670897	1									
*Cis*-Sabinenehydrate	0.283744	0.139118	0.336217	0.103998	−0.1745	1								
γ-Terpinene	−0.1295	−0.32603	0.169215	0.341887	0.410815	−0.0918	1							
*p*-Cymene	−0.49605	−0.66767	0.316183	−0.13416	0.020298	0.127817	0.094421	1						
α-Terpinene	−0.3388	−0.53729	−0.35214	0.125814	0.33052	−0.06435	0.541719	0.065756	1					
Myrcene	0.333103	−0.07724	0.257894	0.521971	0.848305	−0.08688	0.363083	0.071604	0.3681	1				
β-Pinene	0.204731	0.168883	0.031439	0.129113	0.183408	−0.03956	0.296315	−0.2184	0.209804	0.3998	1			
Sabinene	0.233852	−0.1599	0.448399	0.37312	0.646361	0.119777	0.50347	0.087615	0.314928	0.772784	0.369294	1		
α-Pinene	0.29477	−0.05667	0.38278	0.594161	0.768623	0.040513	0.547874	0.091211	0.31989	0.856055	0.505213	0.86658	1	
α-Thujene	−0.08084	−0.40103	0.251448	0.170625	0.550725	−0.12406	0.694547	0.224641	0.433202	0.653886	0.391084	0.838733	0.694635	1

**Table 6:** Correlation between compounds at 90% drought stress.

	Carvacrol	Thymol	Pulegone	Terpinene 4-ol	β- Thujone	Cis- Sabinenehydrate	γ- Terpinene	*p*- Cymene	α- Terpinene	Myrcene	β- Pinene	Sabinene	α- Pinene	α- Thujene
Carvacrol	1													
Thymol	0.741877	1												
Pulegone	0.281354	0.130547	1											
Terpinene 4-ol	0.611854	0.496753	0.213022	1										
β-Thujone	0.537464	0.123386	0.441101	0.693077	1									
*Cis*-Sabinenehydrate	0.266324	0.167935	0.346257	0.086182	0.08395	1								
γ-Terpinene	−0.13422	−0.30373	0.135161	0.252883	0.296684	−0.13576	1							
*p*-Cymene	−0.49418	−0.65629	0.318681	−0.25732	0.069671	0.081158	0.002202	1						
α-Terpinene	−0.36118	−0.52584	−0.35174	0.042078	0.103137	−0.06483	0.520206	0.045548	1					
Myrcene	0.390525	0.031631	0.154068	0.507872	0.732018	0.200358	0.386672	−0.08394	0.454131	1				
β-Pinene	0.221758	0.183315	0.037166	0.216875	0.222119	−0.01403	0.27504	−0.21544	0.192409	0.435785	1			
Sabinene	0.25764	−0.11654	0.425613	0.268726	0.610141	0.167027	0.464974	0.041021	0.316537	0.747977	0.41955	1		
α-Pinene	0.272433	−0.05193	0.366278	0.470676	0.746719	−0.00064	0.536535	0.064597	0.328533	0.788418	0.496061	0.8477	1	
α-Thujene	−0.05261	−0.39416	0.294253	0.055573	0.40886	−0.01281	0.663457	0.225511	0.411003	0.578624	0.441821	0.789627	0.667464	1

**Table 7:** The geographical characteristics of the studied ajowan populations.

No	Accession Number	Location	Accession Code	Geographical Region	Latitude	Longitude	Altitude (m)
3	38,924	Khorasan, Iran	Khorbir	East	32°53′ N	59°13′ E	1461
6	37,483	Mohammadieh, Khorasan, Iran	Khormo	East	32°55′ N	59°13′ E	1460
8	15,226	Khomein, Markazi, Iran	Arakkho	West	33°38′ N	50°4′ E	1811
11	14,322	Hamedan, Hamedan, Iran	Hamdan	West	34°47′ N	48°30′ E	1818
15	15,484	Shahedieh, Yazd, Iran	Yazshah	Center	31°56′ N	54°16′ E	1193
19	943	Fozveh, Isfahan, Iran	Esfahfo	Center	32°36′ N	51°26′ E	1615
18	4077	Ghahderijan, Isfahan, Iran	Esfahgh	Center	32°34′ N	51°26′ E	1615
20	20,055	Qazvin, Qazvin, Iran	Qazvin	North	36°16′ N	49°59′ E	1305
24	17,861	Shiraz, Fars, Iran	Farsfars	South	29°35′ N	52°35′ E	1508
22	10,569	Ardabil, Ardabil, Iran	Ardebil	Northwest	38°16′ N	48°18′ E	1332

## Data Availability

The original contributions presented in this study are included in the article/Supplementary Materials. Further inquiries can be directed to the corresponding author.

## Supplementary Materials

The following supporting information can be downloaded at: https://www.mdpi.com/article/10.3390/foods14010124/s1, Figure S1: The GC-MS chromatogram of some studied ajowan populations under studied treatments.
