# Supplementary figures and images for: Nano Silicon Modulates Chemical Composition and Antioxidant Capacities of Ajowan (Trachyspermum ammi) Under Water Deficit Condition
Source: Foods. 2025 Jan 3;14(1):124. doi: 10.3390/foods14010124 (PMC11719498; doi:10.3390/foods14010124)

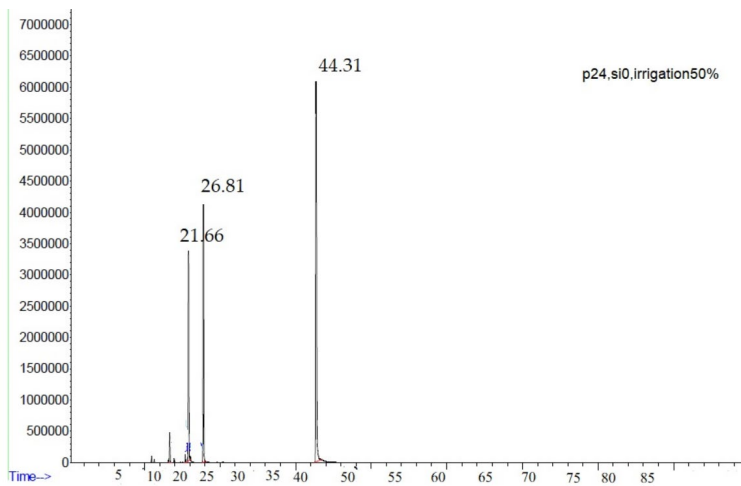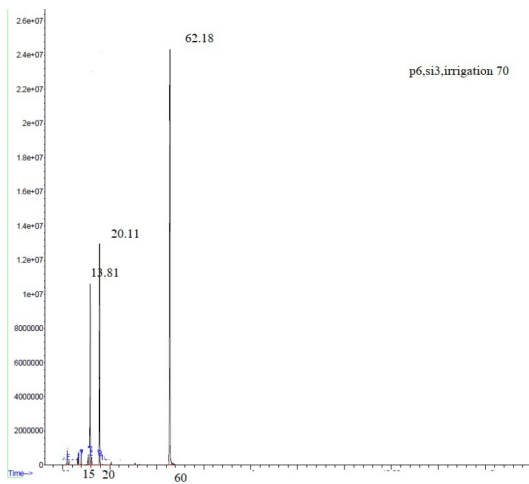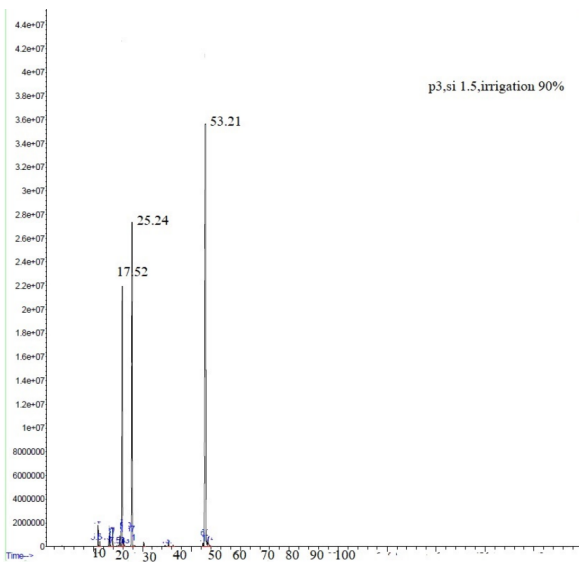

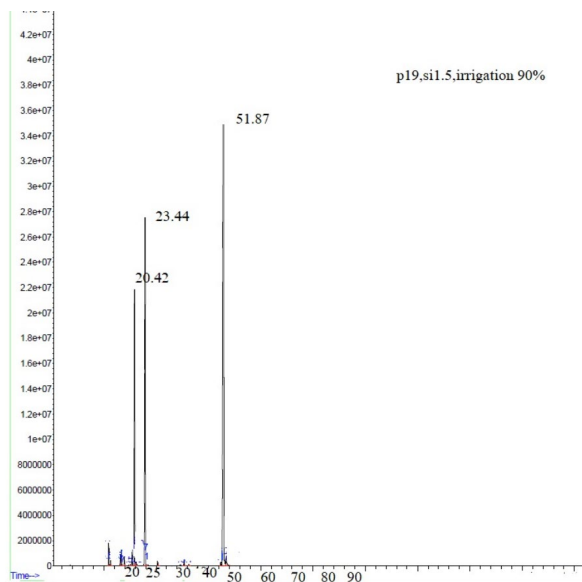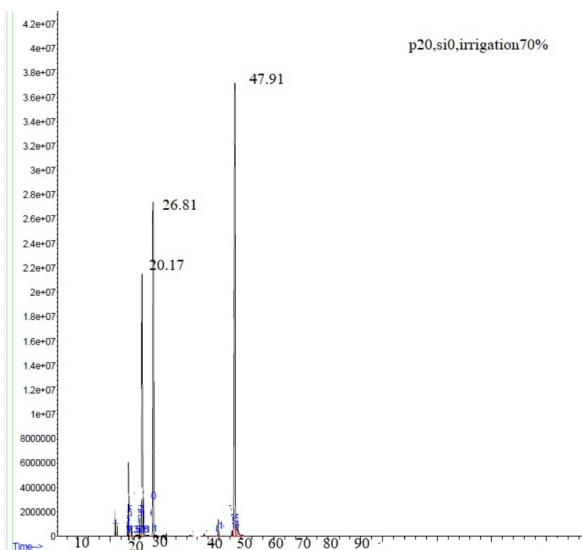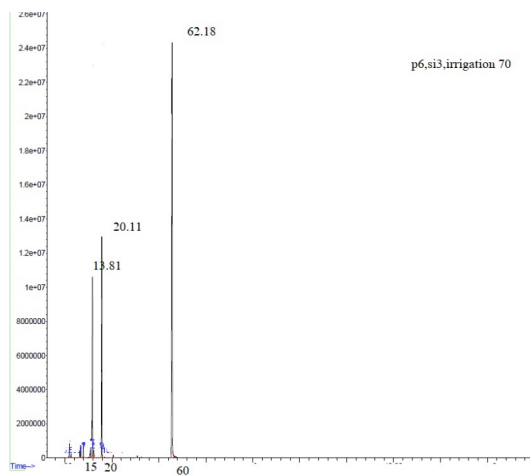

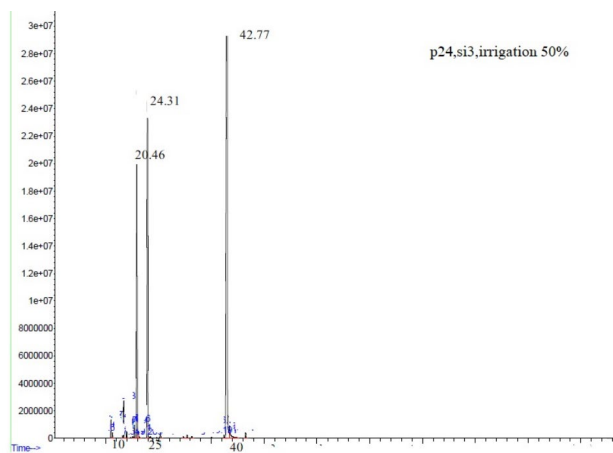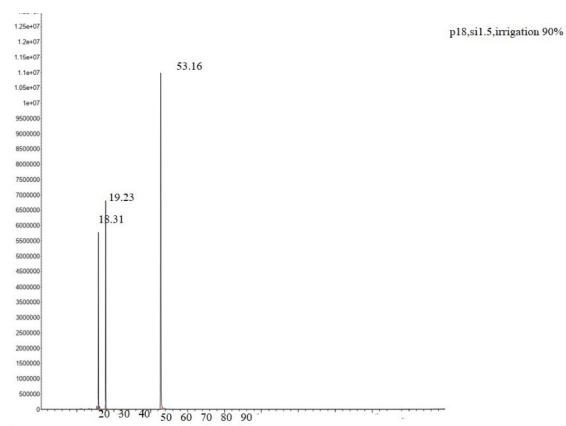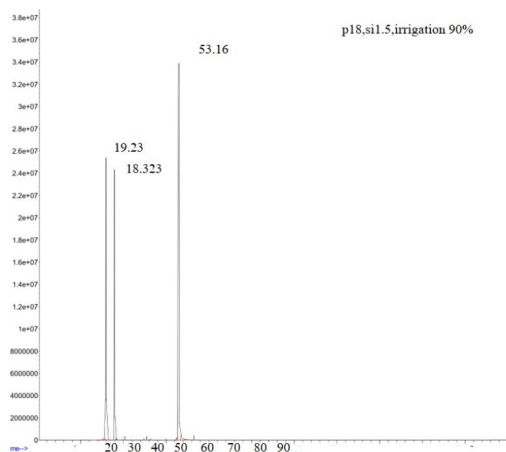

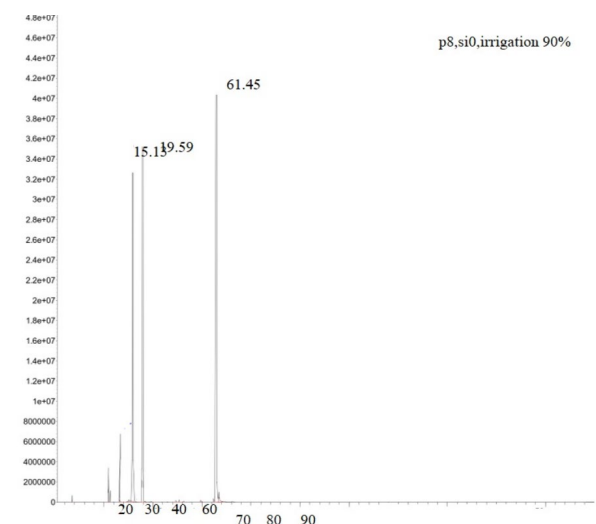

Supplement: Supplementary file 1 [file foods-14-00124-s001.zip › foods-3359717-supplementary.pdf]
